# Conventional health care service utilization among cancer survivors that visit traditional and complementary providers in the Tromsø study: a cross-sectional study

**DOI:** 10.1186/s12913-021-07445-6

**Published:** 2022-01-11

**Authors:** Kiwumulo Nakandi, Dana Mora, Trine Stub, Agnete E. Kristoffersen

**Affiliations:** National Research Center in Complementary and Alternative Medicine (NAFKAM), Faculty of Health Sciences, Department of Community Medicine, UiT The Arctic University of Norway, N-9037 Tromsø, Norway

**Keywords:** Cancer, Health service, Complementary and alternative medicine, CAM, Traditional and complementary medicine, T&CM, Complementary therapies, Norway, The Tromsø study

## Abstract

**Background:**

Traditional and complementary medicine (T&CM) is commonly used among cancer patients worldwide. Cancer patients in Norway mainly visit T&CM providers in addition to conventional health care services. It is not known how their utilization of T&CM providers influences their use of conventional health care services. The aim of this study was to investigate the difference between the utilization of conventional health care services among cancer survivors that visit T&CM providers and those that do not, and their associated factors.

**Method:**

Health care service utilization data were obtained from cancer survivors 40 years and above participating in the Tromsø Study: Tromsø 7 conducted in 2015–2016. Data were collected from self-administered questionnaires. Pearson chi-square tests, Fisher exact tests, t-test, and logistic regression were used, with the significance level considered at *p* < 0.05.

**Results:**

Of 1553 individuals, 10% (*n* = 155) reported visiting T&CM providers in the past 12 months. As both cancer survivors visiting and not visiting T&CM providers were frequent users of conventional health care, no significant differences were found in the overall use of conventional health care (98.1vs.94.5%, *p* = .056). Users of T&CM providers were however more likely to visit physiotherapists (40.1% vs 25%, *p* < .001), emergency rooms (29.2% vs 16.5%, *p* < .001), chiropractors (17% vs 6%, *p* < .001), and psychologist/psychiatrist (8.9% vs 3.4%, *p* < .001). They also had more frequent visits to conventional health care (11.45 vs 8.31 yearly visits, *p* = 0.014), particularly to general practitioners (5.21 visits vs. 3.94 visits, *p* = .002).

**Conclusion:**

Results from this study show that visits to T&CM providers are associated with more visits to conventional health care services among cancer survivors. Further studies are needed to investigate the reasons for this high use behavior.

## Background

Cancer is the second most common cause of death globally [1] and the leading cause of death in Norway [2]. Its incidence is relatively stable, with a small increase each year due to an aging population [3]. By the end of 2019, there were about 300,000 cancer survivors in Norway [4]. Cancer survivorship can be described as the period from diagnosis until the end of life [5]. Almost 3 out of 4 individuals with cancer survive for 5 years or more [6].

Upon cancer suspicion, the general practitioner (GP) who is often the first encounter for patients, will refer the patient to a cancer patient pathway [7]. The pathway contributes to rapid assessment and treatment initiation [8]. Different types of cancer treatments are offered, such as surgery, chemotherapy, radiotherapy, hormonal therapy, stem cell therapy, immune therapies, and palliation [9]. Differences in healthcare models [10] make international comparisons of health service utilization complicated. Norway has universal health coverage, and GP and specialist outpatient consultations are co-paid with a small user fee. Furthermore, the majority of cancer treatments are free for patients [11] and hospital admissions are also free of charge [12].

Following active treatment, cancer survivors have healthcare surveillance needs related to cancer [13–15], cancer treatment [13, 16], and other medical [17] and psychological comorbidities [18, 19]. Post-treatment follow-up is provided by GPs, oncologists, and other specialists, [18, 20] as well as rehabilitation providers such as physiotherapists and chiropractors [21]. Even though cancer survivors were found to have poorer physical [22] and mental health-related quality of life than non-cancer patients [23, 24], studies show that they often do not receive the appropriate follow-up care despite evidence of high-use of services [25–27].

Cancer survivors have on average 7 contacts to specialist health care annually, with some reaching up to 50 contacts. The total number of specialist health service contacts for cancer survivors (admissions, day treatment, outpatient visits) amounted to approximately 139,000 in 2017 [28]. In the same year, the median number of GP contacts was 5 per cancer survivor, varying from 1 to 40 compared to 2.7 contacts by non-cancer patients [17]. Furthermore, cancer survivors are sevenfold more likely to be high-users of out-of-hours centers (medical services for immediate medical assistance [29]) than non-cancer patients [30].

In addition to conventional medical therapies (CM), cancer survivors are increasingly seeking out traditional and complementary medicine (T&CM) [31–33]. The World Health Organization describes traditional medicine as “the sum total of the knowledge, skill, and practices based on the theories, beliefs, and experiences indigenous to different cultures, whether explicable or not, used in the maintenance of health as well as in the prevention, diagnosis, improvement or treatment of physical and mental illness” [34]. Further, complementary medicine is described as “a broad set of health care practices that are not part of that country’s own tradition or conventional medicine and are not fully integrated into the dominant health-care system” [34]. In Norway, provider or non-provider-based T&CM use falls under the alternative treatment act of illness where alternative means, “health-related treatment which is practiced outside the established health services, and which is not practiced by authorized health personnel. However, treatment practiced within the scope of the established health services or by authorized health personnel is also covered by the term alternative treatment when the methods used are essentially methods that are used outside the established health services” [35]. The most common reasons given for T&CM utilization among cancer patients are improvement of physical and emotional wellbeing, as well as strengthening the body’s ability to fight cancer [33]. Earlier studies have shown that factors like gender, higher education, and poorer self-reported health [36] are predictors of T&CM use, while mental health [37], phase of survivorship [38], as well as gender [39], are predictors of high utilization of conventional health care services.

In Europe in general, and in Norway specifically, over a third of cancer survivors reported using some form of T&CM, with small variation across countries [40–42]. The most common therapies are dietary supplements, herbal medicines, and homeopathy, followed by spiritual and relaxation therapies [43–45]. Some Norwegian public hospitals have provisions for T&CM for cancer survivors [46, 47], mainly through the six Varde centers [48] that offer counseling, massage, yoga, dietary advice, and physical activities to cancer survivors and their families [49]. However, most T&CM treatments are offered outside hospitals and are paid for out-of-pocket [50].

Cancer patients in Norway mainly visit T&CM providers in addition to conventional health care services [36]. In a systematic review that looked at T&CM use in Australia, Reid et al. found that users of T&CM had higher use of conventional services [51]. In a study that looked at the health care utilization in general practice patients using T&CM, Kersnik found that they had more use of acute, primary, and secondary health care services [52]. To the authors’ knowledge, conventional health care utilization by cancer survivors that use T&CM providers has not been explored in Norway. The aim of this study is to investigate the difference between utilization of conventional health care services among cancer survivors that visit T&CM providers and those that do not, and their associated factors.

## Method

### The Tromsø study

The data used in this study were abstracted from The Tromsø Study: Tromsø 7. The Tromsø study is an ongoing longitudinal, cross-sectional cohort study that was initiated in 1974 to study cardiovascular risk factors in the Tromsø population. Tromsø is the largest city and municipality in Northern Norway with 73,480 inhabitants at the time of the study [53]. The study consists of 7 surveys to date. The seventh survey was conducted in 2015 – 2016 [54]. Based on the official population registry, inhabitants aged 40 years and above were invited to participate (*n* = 35,591) of whom 21,083 (10,009 men and 11,074 women) agreed to participate yielding an attendance rate of 65% with higher attendance rate among women (67%) than men (62.4%) [54]. Non-attendees tend to be younger and male [55]. Further detailed information about the age and sex distribution according to attendance is also available [55].

**Fig. 1:**
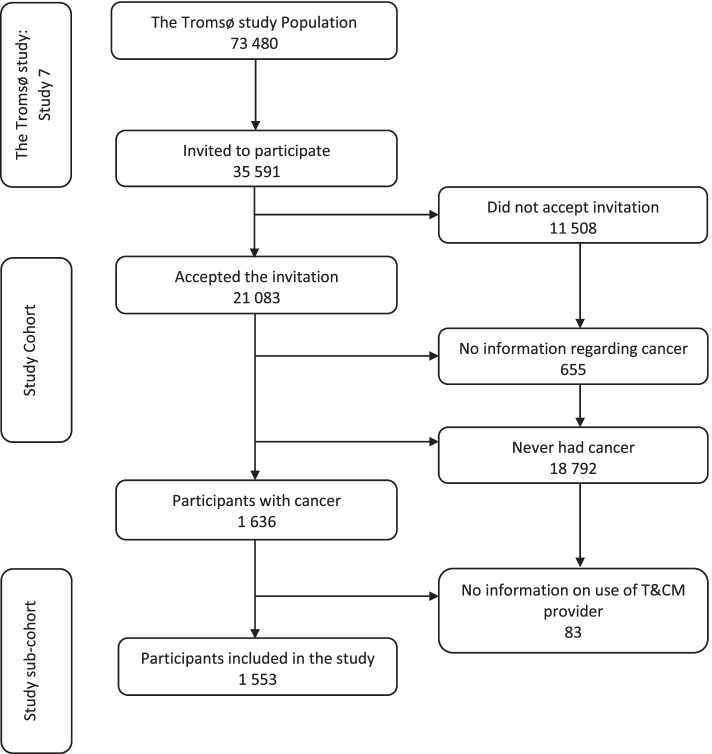
Flowchart of study participants

Eighteen thousand seven hundred ninety-two participants who never had cancer were excluded, as well as 655 participants who did not provide information regarding cancer. A further 83 participants that did not answer any question regarding the use of T&CM providers at all were excluded. The final study sample consisted of 1553 participants who met our inclusion criteria of self-reported present or previous cancer and information on the use of T&CM providers (Fig. 1).

### Data collection

Invitations to participate were sent through postal letters and included a study information brochure and a paper four-page questionnaire, Q1, and a date for a clinical examination. A username and password for a digital version of Q1, and a more comprehensive digital questionnaire (Q2), with expanded questions regarding diseases, health status, socio-economic status, use of health care services, etc., were also included [55].

### Variables

The participants’ basic characteristics, state of health, and use of health care services used in this study were collected from Q1 and Q2.

#### Basic characteristics of participants

Age was measured continuously and reported as mean age with standard deviation (SD) per 31.12.2015. It was then grouped into three groups: “40-60 years”, “61-70 years” and “71 years and above”.

The “male” and “female” categories were collected through the national identity number. The Norwegian national identity number consists of 11 digits, where the ninth digit indicates assigned sex at birth [56].

Living arrangement was assessed through the question “Do you live with a spouse/partner?” with “Yes” and “No” as answer options.

Household income was collected through 7 response categories (“Less than NOK 150’/€ 15’”, “NOK 150’-250’/€ 15’-25’”, “NOK 251’-350’/€ 25.1’-35’”, “NOK 351’-450’/€ 35.1’-45’”, “NOK 451’-550’/ € 45.1’-55’”, “NOK 551’-750’/€ 55.1’-75’”,“NOK 751′-1000′/€ 75.1′-100′” and “more than NOK 1,000’/€ 100’”). These were re-categorized into: “low income” (less than NOK 450′/€ 45′), “middle income” (NOK 450′-750′/€ 45′-75′) and “high income” (more than NOK 750′ /€ 75′).

“What is the highest level of education you have completed?” assessed level of education with 4 categories: “Primary education (up to 10 years of schooling)”, “Secondary education: (a minimum of 3 years)”, “College/university less than 4 years”, “College/university 4 years or more”.

#### Health status

Data about self-reported cancer were collected from Q1 through the question: “Have you ever had, or do you have cancer?” with the alternatives “No”, “Yes, now” and “Previously, not now”.

Self-reported health was measured through a categorical variable with five categories in Q1 and a 100-point Likert scale in Q2. The question from Q1: “How do you in general consider your own health to be?” had the following answer options, “very bad”, “bad”, “neither good nor bad”, “good”, and “excellent”. These were compressed to, “bad” (very bad and bad), “neither good nor bad” and “good” (good and excellent). The question from Q2 stated; “We would like to know how good or bad your health is today” and was measured by a scale numbered from 0 to 100, with 100 representing best possible health.

For assessment of pain/discomfort and anxiety/depression, five categories were used in Q2, where respondents were to mark only one statement that described their health at the time of the survey. For “pain/discomfort” the alternatives were “I have no pain or discomfort”, “I have slight pain or discomfort”, “I have moderate pain or discomfort”, “I have severe pain or discomfort”, and “I have extreme pain or discomfort”. For anxiety/depression their alternatives were, “I am not anxious or depressed”, “I am slightly anxious or depressed”, “I am moderately anxious or depressed”, “I am severely anxious or depressed”, and “I am extremely anxious or depressed”.

Questions on self-reported health, pain/discomfort, and anxiety/depression were taken from a modified quality of life EQ-5D instrument [57].

#### Utilization of health care services

The use of a T&CM provider was based on a “yes” response to either of these three questions: “Have you during the past year visited a traditional healer (helper, “reader”, etc.?)”, “Have you during the past year visited an acupuncturist?” or “Have you during the past year visited a CM provider (homeopath, reflexologist, spiritual healer, etc.?)”.

To each of the questions above, participants were to report the number of times they had visited each provider. Visits to each T&CM provider were summed and presented as “mean overall number of visits to T&CM provider”.

Participants were to answer “yes” or “no” to the questions “Have you during the past 12 months visited; “a general practitioner (GP)”, “emergency room”, “psychiatrist/psychologist”, “another medical specialist than a general practitioner (GP)”, a “psychologist or psychiatrist (not at a hospital)”, “physiotherapist”, or “chiropractor”? Data on hospital visits was gathered through the “yes” or “no” questions; “Have you during the past year been admitted to a hospital?”, “Have you during the past 12 months visited a psychiatric out-patient clinic”, and “Have you during the past 12 months visited other out-patient clinics, (not psychiatric department)”. Where applicable, respondents were to report the number of times they had used each service during the last 12 months and were summed and presented as mean number of visits.

Questions on the use of T&CM providers were taken from the first level of the international CAM questionnaire (I-CAM-Q) which measures T&CM use [58].

“Overall use of conventional health care” was obtained by merging users of general practitioners, emergency rooms (ER), medical specialists, psychologists, psychiatrists, physiotherapists, chiropractors, as well as out-patient clinics and hospital admissions.

Missing data on one or more, but not all, of the health care provider questions, was interpreted as “no/not having visited the particular provider”.

### Statistics and data analysis

Data were summarized using frequencies and descriptive analyses. To calculate differences between cancer survivors that visited T&CM providers and cancer survivors that did not Pearson chi-square tests, and Fisher exact tests were used for categorical values while binary logistic regression and linear regression were used for adjusted values. Continuous variables were analyzed using independent sample t-test with Levene’s test used to access homogeneity of variance in the tested variables. All calculations were conducted using SPSS for Windows (version 26.0, SPSS, Inc., Chicago, IL) and the significance level was considered at *p* < 0.05.

## Results

### Characteristics and associations of the participants

The mean age of the cancer survivors included in the study was 65.33 years. There were significantly more females than males among the cancer survivors who visited T&CM providers than those that did not, 63.9% females and 36.1% males vs 51.4% females and 48.6% males, respectively, *p* = .003 (Table 1). Most participants lived with a spouse/partner (72.6%), with no significant differences between cancer survivors that visited T&CM providers and those that did not (*p* = .437). There were no significant differences found between groups regarding the level of education (*p* = .213) and a marginal difference in household income, but not at a significant level, *p* = .050. However, a significance was found when adjusted for living with a spouse/partner, *p* = 0.048.

**Table 1:** Basic characteristics of cancer survivors that visit T&CM providers and cancer survivors that do not visit T&CM providers

	Total population		Visits T&CM providers		No visits to T&CM providers		***P***-value*
	(*N* = 1553)	%	(*n* = 155)	%	(*n* = 1398)	%	
**Age**							.336
Mean (SD)	65.33 (10.891)		64.53 (11.408)		65.42 (10.833)		435
40-60 years	495	31.9	56	36.1	439	31.4	
61-70 years	520	33.5	51	32.9	469	33.5	
71 years and above	538	34.6	48	31.0	490	35.1	
**Gender**							.003
Women	817	52.6	99	63.9	718	51.4	
Men	736	47.4	56	36.1	680	48.6	
**Living with a spouse/partner**							.437
Yes	1075	72.6	102	69.9	973	72.9	
No	406	27.4	44	30.1	362	27.1	
**Household income**							.050*
Low (< NOK 450′/ € 45′)	475	32.2	60	41.1	415	31.2	
Middle (NOK 451′-750′/€ 45′-75′)	478	32.4	40	27.4	438	32.9	
High (>NOK 751′/€ 75′)	524	35.5	46	31.5	478	35.9	
**Years of Education**							.213
Primary school	450	29.7	47	31.8	403	29.4	
Secondary school	368	24.3	44	29.7	324	23.7	
College/university less than 4 years	298	19.6	26	17.6	272	19.9	
College/university 4 years or more	401	26.4	31	20.9	370	27	

*p = .048 when adjusted for living with spouse/partner

While most of the participants reported middle and high income in both groups, 41.1% reported low income among the cancer survivors who visited T&CM providers compared to only 31.2% among those that did not (Table 1). Most participants (88.4%) reported good health, with a mean score of 71.28 on a 100-point scale where 100 was the best possible health, with no significant differences between the two groups. A Pearson Chi-square test revealed that visitors to T&CM providers were more likely to have cancer currently (cancer at the time of participation, 33.5%) compared to those that did not visit T&CM providers (23.1%). This difference was significant at the *p* = .004 level (Table 2).

**Table 2:** Cancer survivors’ self-reported health and use of health care services

	Total population		Visits to T&CM providers		No visits to T&CM providers		***P***-value
	(*N* = 1553)	%	(*n* = 155)	%	(*n* = 1398)	%	
**Cancer**							
Cancer now	375	24.1	52	33.5	323	23.1	.004^1^
Cancer previously	1178	75.9	103	66.5	1075	76.9	
**Self-reported health (scale 0-100)**							
Mean (SD)	71.28 (17.541)		69.67 (17.11)		71.46 (17.59)		.236^2^
**Self-reported health (n, %)**							.588^3^
Good	889	88.4	77	86.5	812	88.5	
Neither	107	10.6	11	12.4	96	10.5	
Bad	10	1	1	1.1	9	1	
**Level of pain/discomfort (n, %)**							.085^1^
None/slight	438	33.5	31	24.8	407	34.4	
Moderate	796	60.9	85	68	711	60.2	
Severe	73	5.6	9	7.2	64	5.4	
**Feeling anxiety/depression (n, %)**							.014^3^
None/slight	1148	78.5	102	69.4	1046	79.5	
Moderate	301	20.6	43	29.3	258	19.6	
Severe	14	1	2	1.4	12	0.9	
**Visits to T&CM providers**							
Acupuncturist (n, %)	69	4.5	69	46.6	–		
*Mean number of visits to acupuncturist (SD)*	7.24 (10.258)						
Traditional healer (n, %)	59	3.8	59	40.1	–		
*Mean number of visits to traditional healer (SD)*	1,54 (1.206)						
Other CM providers (n,%)	62	4.0	62	41.1	–		
*Mean number of visits to other CM providers (SD)*	4.96 (6.120)						
**Visiting T&CM providers (n,%)**	155	10.0	155	100.0			
***Mean overall visits to T&CM providers (SD)***	5.64 (8.982)						
**Use of conventional health care services last year**							
Seen a GP	1396	90.2	144	93.5	1252	89.6	.150^1^
*Mean number of visits to GPs (SD)*	4.07 (3.758)		5.21 (4.52)		3.94 (3.64)		.002^2^
Been to ER	269	17.7	42	29.2	227	16.5	<.001^1^
*Mean number of visits to ERs (SD)*	1.48 (1.102)		1.73 (1.633)		1.44 (0.966)		.287^2^
Seen a psychologist/psychiatrist	60	4	13	8.9	47	3.4	.001^1^
*Mean number of visits to psychologist/psychiatrist (SD)*	6.37 (7.544)		4.85 (7.255)		6.82 (7.650)		.413^2^
Seen a specialist other than GP	390	26	43	30.1	347	25.6	.241^1^
*Mean number of visits to specialist other than GP (SD)*	2.13 (5.407)		1.90 (1.518)		2.16 (5.699)		.772^2^
Seen a physiotherapist	406	26.4	59	40.1	347	25.0	<.001^1^
*Mean number of visits to physiotherapist (SD)*	11.79 (12.894)		12.40 (16.102)		11.7 (12.363)		.728^2^
Seen a chiropractor	109	7.1	25	17.0	84	6.0	<.001^1^
*Mean number of visits to chiropractors (SD)*	5.79 (8.334)		4.79 (5.051)		6.03 (8.954)		.564^2^
Hospital admissions	376	24.4	48	31.4	328	23.6	.035^1^
*Mean number of admissions to hospitals (SD)*	2.35 (3.756)		2.33 (2.077)		2.35 (3.947)		.961^2^
Out-patient psychiatric clinic	42	3.1	5	3.8	37	3.0	.599 ^3^
*Mean number of visits to out-patient psychiatric clinic (SD)*	6 (13.463)		4.75 (3.862)		6.19 (14.428)		.846^2^
Out-Patient General Clinic	817	54	89	61	728	53.3	.077^1^
*Mean number of visits to out-patient general Clinic (SD)*	3.47 (5.917)		3.74 (5.136)		3.43 (6.012)		.643^2^
**Overall use of conventional health care services**	1472	94.8	152	98.1	1320	94.5	.056^1^
***Mean number of visits to conventional providers (SD)***	8.63 (11.052)		11.45 (13.477)		8.31 (10.698)		.0038^2^
**Overall use of traditional, complementary and conventional health care services**	1475	95	155	100	1320	94.5	.003^1^
***Mean number of visits to traditional, complementary and conventional health care services (SD)***	9.15 (11.848)		16.04 (17.347)		8.31(10.698)		< 0.001^2^

^1^Pearson Chi square test

^2^Independent sample t-test

^3^Fisher’s Exact Test

Though most participants reported none/slight levels of anxiety/depression, there were significant differences between the groups. 30.7% of the cancer survivors with visits to T&CM reported moderate to severe anxiety/depression in comparison to 20.5% with no visits to T&CM providers, *p* = .015. Cancer currently (*p* = .004) and anxiety/depression (*p* = .015) were more common among cancer survivors that visited T&CM providers than those that did not, even though there was similar self-reported health among the two groups, *p* = .633 (Table 2).

### Visits to T&CM providers

10% of all the participants (*n* = 155) reported visiting T&CM providers during the last 12 months, with a mean number of 5.64 visits (median 3, range 1-60). The most frequently visited T&CM providers were acupuncturists, visited by 4.5% of the participants (*n* = 69) with a mean number of visits of 7.24, followed by visits to other CM providers (4.0%, *n* = 62) with a mean number of visits of 4.96. Traditional healers were visited by 3.8% of the participants (*n* = 59) with a mean of 1.54 visits.

### Visits to conventional health care services

Most of the participants (94.8%, *n* = 1472) had visited some form of conventional health care services in the past 12 months, with a mean/median number of visits of 8.63/5 (see Table 2 for the specific therapies).

Cancer survivors that visited T&CM providers were just as likely to have visited conventional health care services as cancer survivors that did not (98% vs. 95% respectively, *p* = .056). However, there was a significant positive correlation between visits to T&CM providers and a high frequency of visits to conventional health care providers, 11.45 vs 8.31 visits, *p* = .004. Those who had visits to T&CM providers were more likely to have visited physiotherapist (40% vs 25%, *p* < .001), emergency room (ER) (29% vs 17%, *p* < .001), chiropractors (17% vs 6%, *p* < .001), and psychologist/psychiatrist (9% vs 3%, *p* = .001). Even though there was no significant difference in having seen a GP, users of T&CM providers reported more frequent visits to GPs, 5.21 vs 3.94 visits, *p* = .002 (Table 2).

### Total use of health care services (traditional, complementary, and conventional health care)

Three cancer survivors reported visits to T&CM providers only. A small group of cancer survivors that did not visit T&CM providers reported no visits to conventional health care services either (*n* = 77, 5.5%), while the majority that did not visit T&CM providers reported utilization of conventional health care services (*n* = 1320, 94.5%). This led to a significant difference in total use of health care services last 12 months among cancer patients that visited T&CM providers and those that did not, 100% vs 94.5% respectively, *p* = .003. Nearly all cancer survivors who visited T&CM providers (98.1%, *n* = 152), reported visits to conventional health care services with a mean number of 5.6 visits to T&CM providers and 11.45 visits to conventional health care services. Consequently, cancer survivors who visited T&CM providers had significantly more health care service visits than those that did not, with a mean of 16.04 vs 8.31 visits, respectively, *p* < .001 (Table 2).

In summary, these results show that visits to T&CM providers were associated with more frequent visits to conventional health services among cancer survivors. These results remained when adjusted for factors associated with higher use of T&CM providers; gender, cancer currently/previously, and anxiety/depression (Tables 1 and 2), (*p* = .010).

## Discussion

This study revealed that visits to T&CM providers were associated with more visits to conventional health care services, particularly visits to walk-in services where a referral from a doctor is not required, but also hospital admissions. However, there were no differences in self-reported health, a driving factor in seeking and utilizing health care services [59], between those visiting T&CM providers and those that did not.

Being female was associated with more visits to conventional health care services among those visiting T&CM providers. Studies on gender differences on the utilization of health care have shown that women have higher health care utilization than men [39]. A 2019 study found that female cancer survivors had more doctor visits and hospital admissions than male cancer survivors [60]. Prior research has also shown that in women with breast cancer, the most diagnosed type of cancer among women [61], side-effects of treatments and impairments may persist up to a decade, influencing women’s use of health care services [62, 63]. However, this only partly explains the differences in health care utilization as the differences remain when adjusted for sex.

Another associated factor of increased visits to conventional health care services among T&CM users was a current cancer diagnosis. Cancer survivors with a current cancer diagnosis have been shown to have higher health care utilization than those not recently diagnosed [38]. Wong et al. found that the number of contacts to different health care providers was generally higher in the first 9 months of diagnosis [64]. Likewise, Low et al. found that a greater period post-diagnosis was associated with lower odds of following up medical appointments [65].

Contrary to other findings [66], visitors of T&CM providers of this study did not report poorer health status. Comparable self-reported health among visitors and non-visitors, and more visits to conventional services among visitors of T&CM providers could lay grounds for interpretation as high-use behavior. However, more cancer survivors visiting T&CM providers reported moderate and severe anxiety/depression. A recent study showed that severe depression was associated with increased visits to specialist health care [37]. Furthermore, depression and anxiety disorders are associated with a higher likelihood of utilization of acute health services like ER visits, hospitalization, and readmission [19, 67]. Our findings reflected similar findings.

Even though chiropractic is part of many countries’ conventional healthcare, [68], including Norway, its principles can resemble those of some complementary therapies based on concepts of holistic health, vitalism, and non-invasive manipulation to restore and preserve health [69]. Likewise, even though physiotherapy is a conventional treatment, some physiotherapists use complementary modalities like acupuncture and massage [70]. It is therefore unsurprising that more cancer survivors that visit T&CM providers visit chiropractors than cancer survivors that do not visit T&CM providers.

Though not exploring causal mechanisms, our findings show an unambiguous relationship between the use of T&CM, its associated use, and higher visitation rates to conventional health care services, in line with previous studies [36, 51, 52, 71–74].

### Strengths and limitations

One of the main strengths of this study is that the data was collected outside a hospital or other health care service setting. This helps bypass the challenge of provider-patient communication, so participants likely disclosed their actual utilization of T&CM providers. For the same reason, cancer survivors who did not use conventional health care services in the last 12 months were able to participate. T&CM providers are grouped in this study, and this minimizes misinterpretation of the concepts. The study not only evaluated the utilization of different health care services, but also frequency of utilization. Although the study was conducted among a Tromsø population, the health care services evaluated, both conventional and T&CM, are available across Norway. Additionally, access to conventional health care was equal among the participants.

The study is not without limitations. Only the “healthiest” cancer survivors likely responded to the Tromsø Study 7 invitation, as shown by their self-reported health. This could affect the number of T&CM provider users participating in this study as poorer health and/or poor prognosis is associated with more use of T&CM providers [75]. The questionnaires used in this study were not validated as a whole but consisted of individual validated parts. The validity of self-reported data as such may be questioned, although self-report has been shown to be a valid estimate of health care utilization [76].

Self-reported cancer was intentionally not validated as authors were interested in participants’ perception of a cancer diagnosis. Even with studies showing high reproducibility and validity of self-reported findings from the Tromsø study, [77, 78], a possibility of a non-medical confirmation of diagnosis remains. There were no specifications of what “cancer now/cancer previously” meant. This could lead to people not choosing the most appropriate category for their phase of cancer, which would impact parts of the data analysis. Recall bias would affect the validity of health care service utilization and the frequency of use, which is integral to our study. However, this would be expected to be equally distributed among the groups and not influence between-group comparison. Prior health care utilization before diagnosis was not evaluated here and could potentially influence the interpretation of the results as a cancer diagnosis could impact utilization of health care services. This study looks at cancer survivors as a somewhat homogenous group. The reader should bear in mind that type [79], location [15], stage, time since diagnosis, and treatment [13–16, 79] lead to different health care needs for the survivor. Therefore, transferability of these findings to a cancer survivor population is affected. The study also only invited individuals 40 years and above. This could have an impact on our findings as age influences the use of health care services [80].

### Implications of the findings

Our findings highlight associations of increased visits to conventional health care services among cancer survivors that visit T&CM providers. As the survivorship period lengthens due to better screening and treatment, future health care service research should focus on reasons for the high use of services. Understanding this group can lead to the development of more appropriate/integrated health care surveillance, to improve health-related quality of life for cancer survivors. This could also prove cost-effective as it can lead to the implementation of preventative measures.

## Conclusion

This study provides an insight into the behavior of cancer survivors and their use of health care services. Cancer survivors that visited T&CM providers had more frequent visits to conventional health care services than those that did not, despite similarities in self-reported health.

## Data Availability

The raw dataset is not available due to Norwegian privacy regulations. Applicants for any data must be prepared to conform to Norwegian privacy regulations. Access to data files can be applied for upon reasonable request at tromsous@uit.no. For more information visit https://uit.no/research/tromsostudy/project?pid=709148.

## Abbreviations

T&CM: Traditional and Complementary Medicine
TM: Traditional Medicine
CM: Complementary Medicine
CAM: Complementary and Alternative Medicine
GP: General Practitioner
ER: Emergency Room
NOK: Norwegian Kroner
€: Euro
SD: Standard Deviation
REK: Regional Committee of Medical and Health Research Ethics
UiT: University of Tromsø
